# Physical inactivity and self-reported depression among middle- and older-aged population in South Asia: World health survey

**DOI:** 10.1186/s12877-017-0489-1

**Published:** 2017-04-28

**Authors:** Ghose Bishwajit, Daniel Peter O’Leary, Sharmistha Ghosh, Sanni Yaya, Tang Shangfeng, Zhanchun Feng

**Affiliations:** 1 Institute of Nutrition and Food Science, University of Dhaka, Dhaka, 1000 Bangladesh; 2 School of Medicine and Health Management, Tongji Medical College, Huazhong University of Science and Technology, Wuhan, China; 3 School of Psychology, Bangor University, Wales, UK; 4 Department of Sociology, University of Dhaka, Dhaka, 1000 Bangladesh; 5 School of International Development and Global Studies, University of Ottawa, Ottawa, Canada

**Keywords:** Depression diagnosis, Physical activity, Self-reported depression, South Asia, World health survey

## Abstract

**Background:**

With the increase in the understanding of the influence of various lifestyle factors such as sedentary behaviour and level of physical activity (PA) on physical and mental health, there has been a growing research interest on how physical inactivity correlates with depressive outcomes across countries. The present study aimed to examine 1) the pattern of engaging in PA among middle- and older-aged population in four South Asian countries, and 2) whether PA is associated with higher prevalence of depression.

**Methods:**

This cross-sectional study is based on country-representative data obtained from WHO’s World Health Survey (WHS). Subjects were 7204 men and women aged above 50 years from Bangladesh, India, Nepal and Sri Lanka, all of which are classified as Low-and-middle-income countries (LMICs) in World Bank reports. Outcome variables were self-ported depression (SRD) and ever being diagnosed with depression. Association between frequency of moderate (MPA) and vigorous physical activity (VPA) and depression was analysed by multivariable regression methods.

**Result:**

Prevalence of self-reported depression was respectively 47.7%, 40.3%, 40.4% and 11.4% in Bangladesh, India, Nepal and Sri Lanka. Prevalence of being ever diagnosed with depression was highest in Nepal (38.7%), followed by India (17.7%), Bangladesh (2.5%) and Sri Lanka (2%). Multivariable analysis shown statistically significant association between PA and diagnosed depression in Bangladesh and India, but not with SRD. In Bangladesh, compared to those who reported engaging in MPA on daily basis, the odds of reporting diagnosed depression were more than five times higher [AOR = 5.512; 95% CI = 1.159–26.21] for those who never took MPA. In India, those never took VPA had 44% higher [AOR = 1.442; 95% CI = 1.046–1.987] odds of being diagnosed with depression compared those who never engaged in VPA.

**Conclusion:**

Lower frequency of vigorous physical activity were significantly associated with higher rates of depression diagnosed. Based on the findings, it is recommendable that health programs targeting mental health among middle- and older-aged population take steps to promote the level of PA within a multi-dimensional depression prevention framework. Longitudinal studies are needed to understand the role of vigorous and moderate physical activity on the onset and intervention of depression among elderly population in the region.

## Background

Globally, depression has emerged as a serious public health concern owing to its association with a wide range of physiological conditions, increased risk of various non-communicable diseases (NCDs) and suicide, all of which in turn are associated with a higher burden of morbidity and mortality [1–3]. Besides its direct health impacts depression also affects academic and workplace productivity, cognitive performance, fulfillment of social and familial roles and quality of life [4, 5]. Despite its growing recognition and many preventive efforts, this prevalence has been rising steadily and is considered a global health priority [4]. According to a study based on International Consortium of Psychiatric Epidemiology (ICPE) Surveys, lifetime prevalence rates of depression range from 8% to 12% in most countries [6]. Another study based on World Mental Health Surveys reported that approximately 6% of people aged 18 years and above have had an episode of depression in the previous year [7]. Currently one of the leading causes of disability in (terms of Years Lived with Disability (YLDs)) and the fourth leading contributor to the global burden of disease [8]; depression is projected to become the second leading cause of disability worldwide by 2020 (second only to cardiovascular disease (CVD)) [9]. The situation is likely to be particularly challenging for developing regions (e.g. South Asia & Sub-Saharan Africa) who share a higher burden of mental disorders with far less funding, research, and infrastructure facilities.

South Asia, which is home to nearly a quarter of the global population (23%), has one-fifth of its psychiatrically ill patients [10]. Despite this noticeably high burden, countries within South Asia have so far been unable to develop an effective mental healthcare delivery system. The major hindrances to the advancement of mental healthcare systems in these countries are: lack of quality data on national prevalence, determinants and risk factors, low mental health literacy, inadequate research and funding priority by governments and lack of adequately trained professionals. Exact regional estimation is not yet available; however, literature review suggests that the rates of depression in some of the countries (India, Pakistan) are among the highest in the world [11, 12]. This comes as no surprise considering the widespread prevalence of the risk factors e.g. ageing, urbanization, unemployment, substance abuse, natural disaster, political unrest. Moreover, countries in this region have been experiencing a steady economic growth with increasing global interconnectedness, which has been accompanied by certain lifestyle and sociodemographic changes predictive of increased susceptibility to mental illnesses [13]. Some noteworthy aspects regarding lifestyle changes and globalization are rising level of stress, dietary transition and reduced physical activity [14].

Though pharmacological treatment is the usual method of intervention for depressive symptoms, there is a growing consensus that non-pharmacological interventions (behavioral modification) such as regular PA can have promising outcomes in alleviating the symptoms. No PA or inadequate PA has been shown to be associated with a variety of chronic conditions (including mental disorders) [15] and is considered the fourth leading risk factor for global mortality causing an estimated 3.2 million deaths per year (6% of total global mortality) [16]. Results from several meta-analyses suggest that exercise has certain antidepressant effects and that engaging in physical activity can have protective effects on developing the symptoms of depression and anxiety [17]. Although the benefits of exercise to the intervention on depressive symptoms are becoming increasingly popular; there is currently insufficient evidence as well as a lack of high quality data to determine the cost-benefit of exercise intervention in depression [18]. This is particularly true for developing countries in South Asia, especially in the context of elderly people. To this regard, we conducted this study by extracting data from the World Health Survey (WHS) of WHO. We studied four South Asian countries namely Bangladesh, India, Nepal and Sri Lanka and included both male and female subjects aged 50 years and above. The objectives were to explore the pattern of physical activity across various demographic and socioeconomic groups in these four countries, and to measure the association between PA and self-reported depression among the middle- and older-aged population.

## Methods

### About the survey

Data for this study were obtained from World Health Survey of WHO conducted between 2002 and 2004. Datasets are available from WHO data repository upon request. The WHS program is operational in 70 countries including four south Asian nations namely Bangladesh, India, Nepal and Sri Lanka. Objectives of the WHO funded survey were to provide reliable and nationally comparable data on a wide range of health and socioeconomic indicators necessary for monitoring the performance and responsiveness of health systems, progress towards public health related goals and enable policy makers regulate policies, strategies and programmes as necessary by supplying evidence based information [18]. The target population were randomly selected (by a two-stage cluster sampling) male and female adults ageing 18 years or over residing in non-institutional settings (e.g. excluding military reservations, or other non-household living arrangements). For those who were in a health institution (e.g. hospital, hospice, nursing home, home for the aged, etc.) at the time of household visit, interview was conducted either in the institution or upon their return to their household if within a period of two weeks from the first visit to the household. The interviews were done face-to-face in the local language using pencil and paper questionnaires. Each interview lasted for approximately sixty minutes depending on the comprehension and literacy level of the respondent. Interviews were conducted by qualified personnel familiar with the local culture, customs and the language. Multistage cluster sampling method was employed to include eligible individuals and the number of individuals selected were 5924 (response rate 94%, Bangladesh, 9977 for India (response rate 97%), 8818 for Nepal (response rate 98%), 6759 for Sri Lanka (response rate 99%). Further details regarding the survey are available elsewhere [19].

#### Outcome

Depression status was the outcome variable in this study. For depression, both ever diagnosed and self-reported (during 12 months) measures were taken into account. Respondents were asked if they were ever diagnosed with depression. The answer was categorized as- Yes/No.

For self-reported depression, respondents were asked- During the last 12 months, have you had a period lasting several days when you felt sad, empty or depressed. Self-reported response categories to these question was- Yes/No.

The explanatory variable of primary interest was physical activity. Two types of PA used in this study were MPA and VPA. VPA was measured by the following question:

MPA was measured by the following question: *Moderate physical activities make you breathe somewhat harder than normal and may include carrying light loads, bicycling at a regular pace, or doubles tennis. Do not include walking. Again, think about only those physical activities that you did for at least 10 min at a time. During the last 7 days, on how many days did you do moderate physical activities*?

*Vigorous activities make you breathe much harder than normal and may include heavy lifting, digging, aerobics, or fast bicycling. Think only about those physical activities that you did for at least 10 min at a time. During the last 7 days, on how many days did you do vigorous physical activities?*

Answer for MPA and VPA ranged from 0 to 7 days, and was categorised followingly: never = 0 days, 1–2 days, 3–4 days, 5–6 days, and every day.

Based on literature review and availability on the datasets, the other explanatory variables included in the study were- Age: 50–59/60+ years; Sex: Female/Male; Currently married: No/Yes; Educational attainment: Nil/Less than primary school/Primary complete/Secondary complete/High school/equivalent complete/Pre-university/University; Employment status: Government employee/Private employee/ Employer/Unemployed; Smoking habit: Daily/Yes but not daily/Non-smoker; Ever drank alcohol: Yes/No.

### Statistical analysis

Inclusion criteria of the subjects were ageing 50 years or above, availability of information on PA status and all the covariates included in the study. Sample characteristics were analysed using simple descriptive statistics e.g. frequencies and percentages. Cross tabulation was performed to measure the crude prevalence of self-rated health (SRH) status, and the distribution of outcome variables across the sociodemographic variables separately for all four countries. Significance of group differences (Good Vs poor SRH) for the explanatory variables were tested by chi-square tests and was presented as *p*-values. Final step was logistic regression analysis that assessed the adjusted associations of depression with MPA and VPA. Only the variables that had a *p*-value below 0.25 in the cross-tabs were selected for the regression analysis [20]. Four separate regression models were run for each country with those reporting no MPA and VPA as the reference group. The outcomes of the regression analysis were reported in terms of adjusted odds ratios (AOR) and corresponding 95% confidence intervals. Analyses were performed with SPSS version 21 and Stata version 12.

## Results

### Basic sample characteristics

Sample population included 619 men and 589 women from Bangladesh, 1077 men and 1047 women from India, 963 men and 917 women from Nepal, and 858 men and 785 women from Sri Lanka (not shown in the analysis).

Table 1 shows the baseline characteristics of the sample population. Majority of the participants were below 60 years old for all four countries, and male population was more numerous than female except for Sri Lanka. Percentage of population being currently married were was 69.3%, 73.8%, 72.6% and 70.4% for Bangladesh, India, Nepal and Sri Lanka respectively. Literacy rate was highest for Sri Lanka (90%) and lowest for Nepal (16.2%). Bangladesh had the highest unemployment rate (54.5%) followed by India (53.3%) and Sri Lanka (51.1%). Prevalence of ever drinking alcohol (40.1%) and daily smoking (51.3%) was highest in Nepal. Lowest rate of daily smoking was observed in Sri Lanka (17.8%) and alcohol drinking in Bangladesh (9.3%). Percentage of population who reported never engaging in moderate physical activity was highest in Nepal (66%) followed by Bangladesh (64.9%), Sri Lanka (26.1%) and India (26%), and that for vigorous physical activity was highest for Sri Lanka (57.0%) followed by India (55.4%), Bangladesh (38.7%) and Nepal (23.4%).

**Table 1:** Basic sample characteristics. World Health Survey 2002–03

Variables	Bangladesh	India	Nepal	Sri Lanka
Age, Mean(SD)	60.72 (9.6)	59.94 (9.1)	60.75 (9)	60 (9.08)
50–59	50.6	48.8	47.8	54.9
60–69	29.5	33.6	32.2	27.3
70+	20.0	17.7	20.1	17.8
Sex				
Female	48.8	49.0	48.8	52.2
Male	51.2	51.0	51.2	47.8
Currently married				
No	30.7	26.3	27.4	29.6
Yes	69.3	73.8	72.6	70.4
Educational attainment				
Nil	58.4	52.2	83.8	10.0
Primary	30.1	25.4	11.3	45.0
Secondary	7.9	14.8	4.2	42.7
Pre-university/University	3.6	7.6	0.7	2.3
Job				
Govt. employee	3.1	4.1	2.0	6.9
Private employee	3.6	6.5	0.7	7.0
Employer	38.7	36.1	58.3	35.0
Not working for payment	54.5	53.3	39.1	51.1
Smoking habit				
Daily	37.8	42.3	51.3	17.8
Yes, not daily	3.9	3.3	5.9	10.0
Non-smoker	58.3	54.5	42.8	72.2
Ever drank alcohol				
Yes	9.3	11.5	40.1	20.5
No	90.7	88.5	59.9	79.5
Days of moderate physical activity				
0	64.9	26.0	66.0	26.1
1–2	10.0	5.8	6.9	8.4
3–4	7.3	7.6	7.4	11.1
5–6	5.0	12.3	3.4	11.6
7	12.7	48.2	16.3	42.8
Days of vigorous physical activity				
0	38.7	55.4	23.4	57.0
1–2	14.7	6.5	4.7	7.4
3–4	11.5	5.4	5.7	8.1
5–6	8.9	11.6	5.6	9.2
7	26.2	21.1	60.6	18.3

### Prevalence of self-reported depression

Table 2 indicates that prevalence of self-reported depression was respectively 47.7%, 40.3%, 40.4% and 11.4% in Bangladesh, India, Nepal and Sri Lanka respectively. Those who reported suffering from depression were more likely to be ageing below 60 years, female, currently unmarried, having no formal education, unemployed, smoking tobacco on daily basis and ever drinking alcohol. Depression also tended to be more prevalent among those who reported not engaging in any type of physical activity.

**Table 2:** Percentage of population reporting depression in past 12 months across the explanatory variables, World Health Survey 2002–03

Variables	Bangladesh (*n* = 576, 47.7)	India (*n* = 887, 40.3)	Nepal (*n* = 759, 40.4)	Sri Lanka (*n* = 187, 11.4)
Age				
50–59	46.7	47.8	42.7	46.5
60–69	29.9	33.2	33.7	29.9
70+	23.4	19.0	23.6	23.5
*p*	0.007	0.389	<0.0001	0.012
Sex				
Female	56.4	52.4	56.1	58.8
Male	43.6	47.6	43.9	41.2
*p*	<0.0001	0.004	<0.0001	0.032
Currently married				
Yes	37.5	30.1	36.5	38.5
No	62.5	69.9	63.5	61.5
*p*	<0.0001	<0.0001	<0.0001	0.004
Educational attainment				
Nil	62.7	55.2	88.9	50.8
primary school	28.6	26.8	7.5	12.8
Secondary complete	6.6	12.3	3.3	33.7
Pre-university/University	2.1	5.7	0.3	2.7
*p*	0.003	0.001	<0.0001	0.059
Job				
Govt. employee	4.9	3.2	1.8	4.8
Private employee	4.6	6.3	0.7	4.8
Employer	42.4	32.5	54.4	38.0
Not working for payment	48.1	58.0	44.1	52.4
*p*	<0.0001	<0.0001	0.002	0.331
Smoking habit				
Daily	59.3	51.8	41.9	71.1
Yes. not daily	3.5	3.0	6.5	7.0
Non-smoker	37.2	45.3	51.6	21.9
*p*	0.615	<0.0001	0.624	0.130
Ever drank alcohol				
Yes	10.9	11.8	38.6	17.1
No	89.1	88.2	61.4	82.9
*p*	0.035	0.388	0.157	0.129
Days of moderate physical activity				
0	67.5	27.5	68.1	30.5
1–2	9.2	5.5	5.9	10.2
3–4	7.1	7.1	7.1	11.8
5–6	4.3	13.3	2.6	8.0
7	11.8	46.6	16.2	39.6
*p*	0.432	0.383	0.278	<0.0001
Days of vigorous physical activity				
0	43.1	54.8	23.5	62.0
1–2	14.4	6.0	5.0	11.2
3–4	10.8	6.2	4.9	9.1
5–6	8.0	12.4	6.6	6.4
7	23.8	20.5	60.1	11.2
*p*	<0.0001	0.523	<0.0001	<0.0001

Table 3 indicated that the prevalence of being ever diagnosed with depression was highest for Nepal (38.7%), followed by India (17.7%), Bangladesh (2.5%) and Sri Lanka (2%). Similar to SRD, those who were diagnosed with depression were more likely to belong to the youngest age group, female (except for Nepal), currently unmarried, having no formal education and employed, smoked tobacco on daily basis and ever drank alcohol, and did not engage in any type of physical activity.

**Table 3:** Percentage of population ever been diagnosed with depression across the explanatory variables, World Health Survey 2002–03

Variables	Bangladesh (*n* = 30, 2.5)	India (*n* = 413, 17.7)	Nepal (*n* = 727, 38.7)	Sri Lanka (*n* = 33, 2)
Age Mean				
50–59	60.0	47.0	49.9	66.7
60–69	23.3	35.8	30.7	15.2
70+	16.7	17.2	19.4	18.2
*p*	0.377	0.484	0.324	0.262
Sex				
Female	56.7	53.0	48.6	63.6
Male	43.3	47.0	51.4	36.4
*p*	0.241	0.1	<0.0001	0.125
Currently married				
Yes	23.3	28.8	26.3	24.2
No	76.7	71.2	73.7	75.8
*p*	0.132	0.213	0.208	0.318
Educational attainment				
Nil	40.0	55.0	82.7	15.2
Primary school	46.7	25.9	11.1	30.3
Secondary complete	3.3	12.6	5.2	51.5
Pre-university/University	10.0	6.5	1.0	3.0
*p*	0.032	0.471	0.218	0.362
Job				
Govt. employee	6.7	2.9	2.1	3.0
Private employee	0	8.0	1.1	6.1
Employer	40.0	33.2	59.8	42.4
Not working for payment	53.3	55.9	37.0	48.5
*p*	0.502	0.158	0.116	0.715
Smoking habit				
Daily	30.0	34.4	50.1	12.1
Yes, not daily	10.0	1.0	4.7	6.1
Non-smoker	60.0	64.6	45.3	81.8
*p*	0.177	<0.0001	0.082	0.465
Ever drank alcohol				
Yes	16.7	12.1	40.7	15.2
No	83.3	87.9	59.3	84.8
*p*	0.137	0.422	0.338	0.301
Days of moderate physical activity				
0	63.3	23.5	62.9	24.2
1–2	10.0	6.5	7.4	3.0
3–4	10.0	9.0	8.7	9.1
5–6	3.3	12.8	3.3	9.1
7	13.3	48.2	17.7	54.5
*p*	<0.0001	0.183	0.189	0.1
Days of vigorous physical activity				
0	36.7	58.4	21.7	51.5
1–2	23.3	7.7	4.5	9.1
3–4	20.0	5.6	5.9	15.2
5–6	13.3	10.2	6.1	6.1
7	6.7	18.2	61.8	18.2
*p*	0.070	0.227	0.123	0.030

### Association between PA and SRD

Results of multivariable regression on the association between frequency of PA and SRD were presented in Table 4. Results indicated no statistically significant association between MPA and VPA with self-reported depression during last 12 months. However, compared to those who took moderate PA on daily basis, the odds of reporting depression were respectively 28% [AOR = 1.284; 95% CI = 0.938–1.758], 34% [AOR = 1.336; 95% CI = 0.873–2.046] and 13% [AOR = 1.125; 95% CI = 0.754–1.678] higher among those who never engages in PA in Bangladesh, India and Sri Lanka. With regard to diagnosed depression, compared to those who reported engaging in MPA on daily basis, the odds of reporting depression were more than five times higher [AOR = 5.512; 95% CI = 1.159–26.21] for those who never took MPA in Bangladesh (Table 5). In India, those never took VPA had 44% higher [AOR = 1.442; 95% CI = 1.046–1.987] odds of being diagnosed with depression compared those who never engaged in VPA.

**Table 4:** Association between PA and self-reported depression during last 12 months, World Health Survey 2002–03

	Bangladesh	India	Nepal	Sri Lanka
Days of MPA				
7	-	-	-	-
5–6	1.034 (0.712–1.500)	1.155 (0.912–1.462)	0.728 (0.449–1.181)	1.639 (0.870–3.088)
3–4	1.140 (0.760–1.710)	1.334 (0.907–1.961)	1.066 (0.670–1.695)	1.135 (0.647–1.993)
1–2	1.135 (0.726–1.774)	0.953 (0.668–1.359)	0.608 (0.386–0.957)	1.044 (0.578–1.885)
0	1.284 (0.938–1.758)	1.336 (0.873–2.046)	0.969 (0.672–1.123)	1.125 (0.754–1.678)
Days of VPA				
7	-	-	-	-
5–6	1.086 (0.722–1.635)	0.990 (0.670–1.462)	1.108 (0.742–1.652)	0.545 (0.314–0.944)
3–4	0.871 (0.545–1.390)	0.660 (0.437–0.997)	0.964 (0.659–1.410)	0.775 (0.432–1.390)
1–2	1.099 (0.631–1.912)	0.895 (0.644–1.242)	1.056 (0.771–2.385)	1.292 (0.669–2.494)
0	0.955 (0.653–1.396)	0.943 (0.732–1.214)	0.987 (0.668–1.176)	1.592 (0.941–2.693)

N.B. Adjusted for variables found significant at *p* < 0.25 in the bivariate analysis. *MPA* moderate physical activity, *VPA* vigorous physical activity

**Table 5:** Association between PA and being ever diagnosed with depression, World Health Survey 2002–03

	Bangladesh	India	Nepal	Sri Lanka
Days of MPA				
7	-	-	-	-
5–6	0.729 (0.261–2.041)	0.687 (0.410–1.153)	1.011 (0.624–1.639)	0.807 (0.326–2.0)
3–4	0.548 (0.186–1.619)	0.622 (0.392–0.986)	0.926 (0.592–1.449)	1.524 (0.364–6.384)
1–2	0.716 (0.210–2.439)	0.629 (0.405–0.979)	0.838 (0.535–1.314)	1.360 (0.329–5.616)
0	5.512 (1.159–26.21)	0.928 (0.540–1.981)	0.942 (0.731–1.215)	3.125 (0.372–26.261)
Days of VPA				
7	-	-	-	-
5–6	0.956 (0.261–3.501)	0.997 (0.631–1.574)	0.853 (0.582–1.249)	0.584 (0.156–2.191)
3–4	0.667 (0.178–2.496)	1.167 (0.699–1.947)	0.725 (0.502–1.048)	0.363 (0.120–1.101)
1–2	1.104 (0.135–9.055)	1.487 (0.977–2.263)	0.962 (0.564–1.642)	1.041 (0.221–4.898)
0	0.553 (0.169–1.812)	1.442 (1.046–1.987)	0.797 (0.604–1.052)	0.964 (0.345–2.698)

N.B. Adjusted for variables found significant at *p* < 0.25 in the bivariate analysis. *MPA* moderate physical activity, *VPA* vigorous physical activity

## Discussion

### Main findings

Findings indicate that the prevalence of self-reported depression (SRD) during the previous 12 months was highest in Bangladesh and lowest in Sri Lanka. Almost two-fifth of the subjects reported suffering from depression in India and Nepal. As expected, prevalence of ever being diagnosed with depression was lower than SRD. Prevalence of ever being diagnosed with depression was highest in Nepal and lowest in Sri Lanka. Surprisingly, Bangladesh also had a low rate of diagnosed depression despite the highest prevalence of SRD. Both SRD and diagnosed depression were more prevalent among the youngest age group, female, unmarried subjects. Those who were unemployed and had the habit of smoking and drinking were more likely to report both SRD and diagnosed depression. Prevalence of regular moderate physical activity (MPA) was highest in India and lowest in Bangladesh, and that of vigorous physical activity (VPA) was highest in Nepal and lowest in Sri Lanka.

In the multivariable analysis, no statistically significant association was observed between SRD and PA of any kind. However, compared to engaging in MPA on a regular basis (seven days a week), zero days of MPA showed higher odds of SRD in Bangladesh and India, but not in Nepal and Sri Lanka. Regarding diagnosed depression; statistically significant association with MPA was observed for Bangladesh, and VPA for India. In Bangladesh, never engaging in MPA increased the odds of diagnosed depression by more than five times and in Sri Lanka by more than three times (compared to those who exercised MPA on a regular basis).

### Comparison with existing studies

Some major challenges for comparing the studies on the prevalence of depression and PA are the variations in target groups and the way these variables are defined. A systematic review on the patterns physical activity among South Asian adults reported that the prevalence of inactivity was of 18.5%–88.4% in India and 11.0%–31.8% Sri Lanka [21]. At the time of writing, the current authors could find no studies of nationwide prevalence of depression among the elderly population in South Asia. However, evidence from research at sub-national levels reported that 3–4% of the total population suffers from major mental disorders and 7–10% from minor depressive disorders in India [22]. In a review study, the percentage of the elderly population in India with depression was found to be significantly higher compared to global prevalence (18.2% Vs 5.4%) [23]. A more recent study reported that prevalence rates of depression in India range from 1.5/1000 to as high as 37.74/1000 [24]. Regional prevalence was reported to be ranging from 6.6% to 11.2% in Sri Lanka, 8–8.9% in Bangladesh to 16.1%–34% in Nepal [25–27].

Regarding the association between PA and depression outcomes; an increasingly large volume of studies encourage physical exercise interventions for the reduction of depressive symptoms. In a literature review including 48 studies, 34 reported a significant reduction of depressive symptoms due to exercise interventions [28]. A cross-sectional study in of the elderly population (aged 60 and above) in Karachi, Pakistan reported a strong association between depression and time spent both in PA and daily living activities. This study suggested a positive role of higher functional status and physical activity in prevention of depression in the elderly [29]. A Spanish study on the elderly population reported that physical activity was related to decreased depressive symptoms among both community-dwellers and institutionalized older adults [30]. In addition, adults practicing the highest levels of physical and athletic activities were at lower risk of developing depression [31].

These discussions indicate that regular PA can be beneficial not only for clinically diagnosed patients, but also can decrease the incidence of depression among the elderly population. Elderly individuals living with depression tend to have a higher morbidity and mortality compared to those without depression. According to a WHO report, older people in South-East Asia with depression had a four times higher rate of mortality compared to those without depression [22]. Our findings suggest that interventions aimed at promoting MPA and VPA may reduce the burden of depression among elderly South Asians.

### Strength and limitations

To our knowledge, this is the first study to explore the association between PA and depression among the middle- and older-aged population in South Asia. The findings can assist health policy makers, and future researchers studying depression among the elderly population. Another strong point of this study was that it included both subjective and objective measures of depression. However, there are certain limitations which should be taken into consideration while interpreting the findings. This was a cross-sectional study and data was collected at the same point in time, therefore no causal inference can be made from the findings. Secondly, as the datasets are old, the prevalence of PA and SRD might have changed. Thirdly, given the secondary nature of the data, we had no control over the measurement and selection of variables which have caused omission of environmental and culturally relevant factors that affect uptake of PA. Future studies may include a broader range of risk factors across different socioeconomic groups to provide a more comprehensive distribution of risk profiles. Finally, as PA was self-reported, there remains a risk of recall and reporting bias by the participants.

## Conclusion

The findings of the study indicated that lower frequency of moderate and vigorous physical activity were associated with higher rates of depression among middle- and older-aged men and women in Bangladesh, India, Nepal and Sri Lanka. Prevalence of depression was highest in Bangladesh and lowest in Sri Lanka. Future research should explore the causes of regional variation and investigate the roles of vigorous and moderate physical activity on the onset and intervention of depression among the elderly population. It is worth mentioning that mental illnesses remain a vastly neglected area of population health research in all South Asian countries. This is a critical concern for population health; particularly for the vulnerable groups such as elderly people who usually suffer multiple medical conditions and have less resources to spend on healthcare. The study concludes with the recommendation for health policy researchers that programs targeted at improving mental health among the elderly population should focus on promoting physical activity within a multi-dimensional framework of prevention of depressive disorders.

## Abbreviation

MPA: Moderate physical activity
SRH: Self rated health
VPA: Vigorous physical activity
WHO: World Health Organization
WHS: World Health Survey
